# Endoscopic treatement by vaginoscopy of a Herlyn-Werner-Wunderlich syndrome: A case report

**DOI:** 10.1016/j.ijscr.2024.109559

**Published:** 2024-03-19

**Authors:** Farah Flissate, Hounaida Mahfoud, Youssef Essebagh, Najia Zeraidi, Amina Lakhdar, Aziz Baidada

**Affiliations:** Gynaecology-Obstetrics and Endoscopy Department, Maternity Souissi, University Hospital Center IBN SINA, University Mohammed V, Rabat, Morocco

**Keywords:** Herlyn-Werner-Wunderlich syndrome, Hematocolpos, Obstructed vagina, Pelvic pain, Vaginoscopy, Septum excision.

## Abstract

**Introduction:**

Herlyn-Werner-Wunderlich syndrome is a rare complex congenital disorder, presenting with obstructed vagina, uterus didelphys and ipsilateral renal agenesis.

Hemivaginal obstruction firstly asymptomatic, leads to symptoms after menarche such as dysmenorrhea, pelvic pain or infertility.

**Case presentation:**

A 15-year-old patient presenting with few symptoms, transvaginal ultrasound reveals an hematocolpos, we report also typical findings of this disorder on magnetic resonance imaging.

**Discussion:**

The pelvic pain caused by the hematocolpos is the main symptom that leads patients to consult often urgently, the MRI is the gold standard exam to confirm diagnosis, the treatment consists on incision the septum vaginal and leads to avoid complications.

**Conclusion:**

Early diagnosis of this syndrome usually leads to initiate surgical treatment in order to avoid complications.

## Highlights

• Herlyn-Werner-Wunderlich Syndrome is a rare Mullerian malformation.
• It's mostly asymptomatic until the onset of the first menstrual period.
• Radiological exams help characterize and describe the malformation's anatomy.
• Early surgical management prevents later complications

## 1 Introduction

Herlyn-Werner-Wunderlich syndrome (HWWS) or known as OHVIRA (Uterus didelphys associated with obstructed Hemi-Vagina and Ipsilateral Renal 1nomaly) is a rare malformation of Müllerian and mesonephric origin. [[1], [2], [3]]

The incidence of this rare symptom is 1/1000.

The diagnosis of this syndrome is evoked in the presence of uterus didelphys, unilateral blind hemivagina and ipsilateral renal agenesis, the common embryological development of the urinary and genital systems explains the associated malformations of both systems. [2]

In some cases, the patient may remain asymptomatic and symptoms as pelvic pain, dysmenorrhea and a pelvic mass may appear a few months after the first menstrual period. In other cases, the diagnosis may be made in the presence of complications like endometriosis and infections. [3]

While pelvic ultrasound can be used to suggest the diagnosis of Hematocolpos, MRI can be used to describe the type of anatomical malformation and confirm the diagnosis. [4]

Full resection of the vaginal septum by endoscopy or by surgery is the treatment recommended, the delay between diagnosis and treatment must be as short as possible, both to relieve the patient and to avoid complications.

We report the case of Herlyn-Werner-Wunderlich syndrome, diagnosed in young female patient with an intact hymen treated by vaginoscopy.

## 2 Case report

This is a 15-year-old girl who was referred to our structure, with no previous history who had consulted for pelvic pain over the past 3 months, which had been progressive and of moderate intensity. The young girl had her period at the age of 13, her cycle was regular, she did not have dysmenorrhea and did not report urinary or digestive signs, her secondary sexual characteristics were well developed.

Physical examination showed normal vulva with the whole hymen and a pelvic mass slightly painful. Abdominopelvic ultrasound images revealed a cystic mass.

**Fig. 1:**
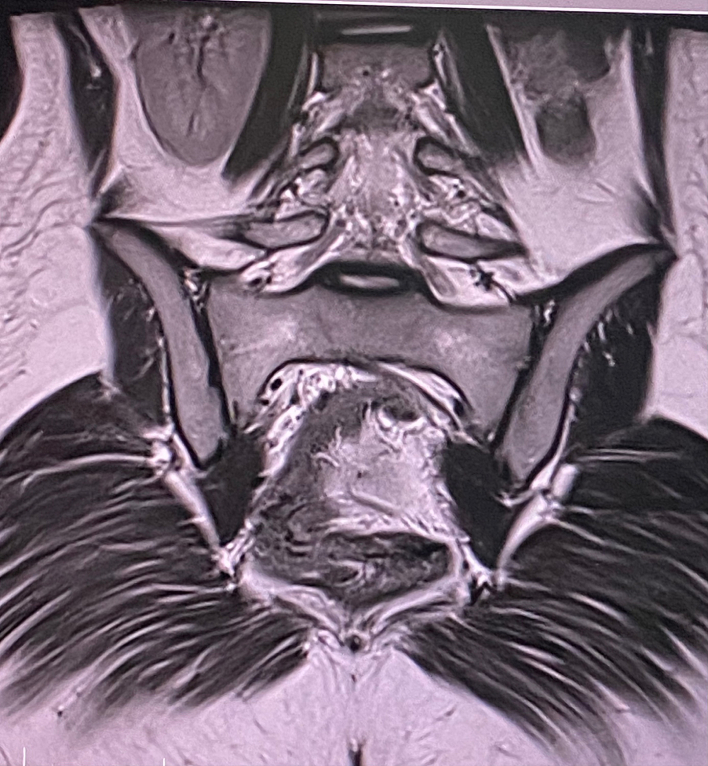
coronal section of IRM showing left renal agenisis.

**Fig. 2:**
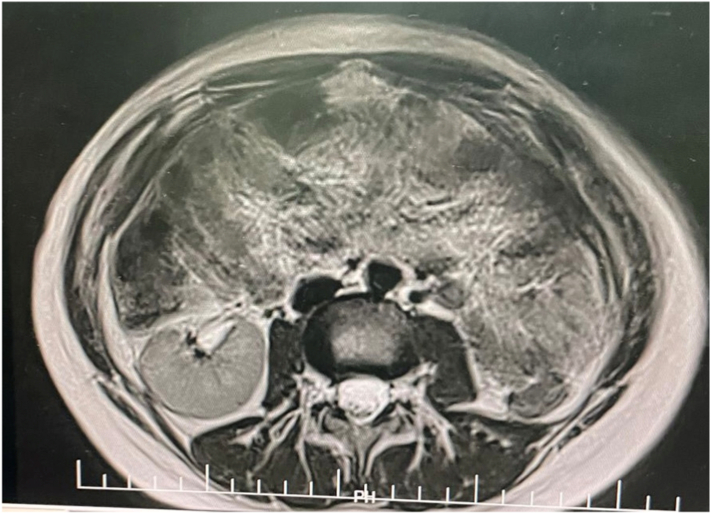
an axial cross-section of IRM showing the agenisis of the left kidney.

**Fig. 3:**
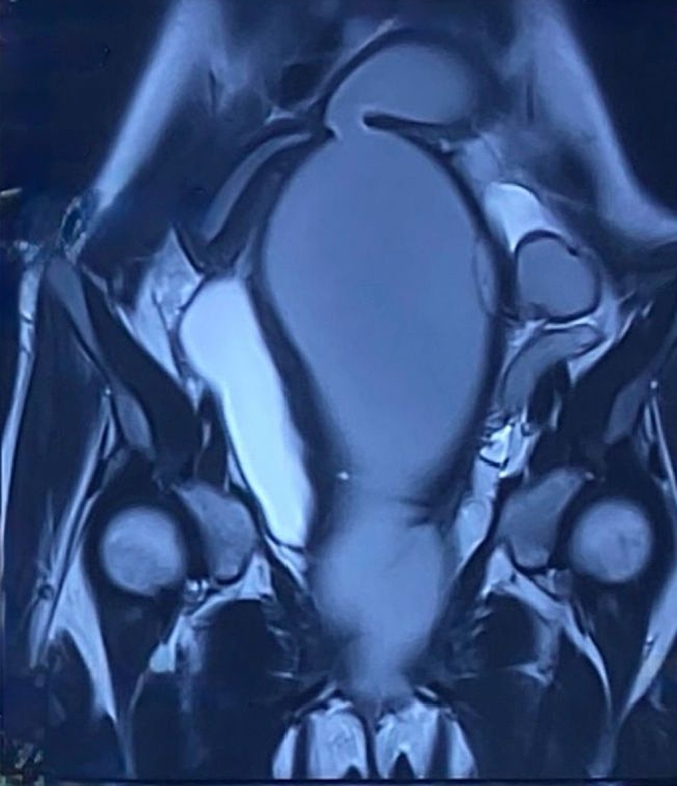
a coronal section of IRM showing a uterus didelphys a left hematometra and a left Hematocolpos.

MRI was performed in three planes in T1 T2 diffusion and Gadolinium injection sequence. The exam confirmed agenesis of the left kidney (Fig. 1, Fig. 2), the existence of two separate uterine bodies and two separate cervices, with obstructed left Hemivagina and a huge left hematocolpos measuring 105*80*210 mm and left hematosalpinx that confirming the presence of uterus didelphys evoking the syndrome of Herlyn Werner Wunderlich. (Fig. 3) The both ovaries were normal.

The surgical procedure lasted 1 h, the patient was under general anesthetic in the gynecological position and a urinary catheter was inserted.

When the patient has no sexual activity, it is recommended to try to preserve the hymen, we did not use a speculum.

The first stage of the operation consisted of a very precise examination, followed by the installation of the operating vaginoscopy to explore the two hemivaginas. We found an enormous hematocolpos bulging in the right vagina.

**Fig. 4:**
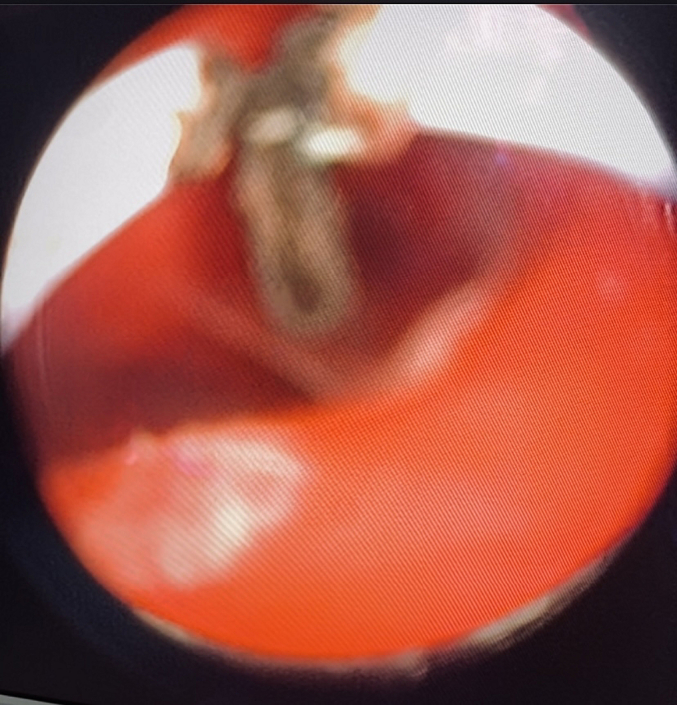
an image of the Vaginoscopy procedure after resection of the vaginal septum showing 2 hemivaginas.

Vaginoscopy incision of the vaginal septum was performed successfully with the monopolar cautery using Collins knife, and the left hematocolpos was evacuated, we then washed abundantly with physiological serum. (Fig. 4).

The left cervix was then clearly visualized.

Reflux urine was visualized during the cystoscopy.

The hymen was not injured during the surgery.

**Fig. 5:**
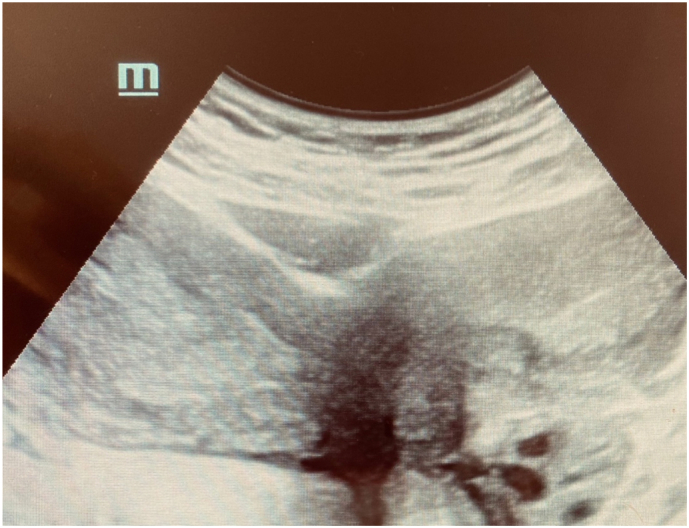
an image of ultrasound showing the uterus didelphys 3 months after surgery.

Postoperative recovery was without complications. A Pelvic ultrasound was done 3 months later and showed 2 uterine cavities, there was no recurrence of hematocolpos. (Fig. 5).

## 3 Discussion

Purslow reports the first case of this syndrome in 1922. [1]

Herlyn-Werner-Wunderlich syndrome, also known as obstructed hemivagina and ipsilateral renal anomaly syndrome, is a complex, rare Müllerian anomaly. [5]. HWWS is distinctive by uterus didelphys, unilateral blind hemivagina, and ipsilateral renal agenesis. HWWS is a rare syndrome with a reported prevalence of around 2 %–3 %, [3,6].

**Fig. 6:**
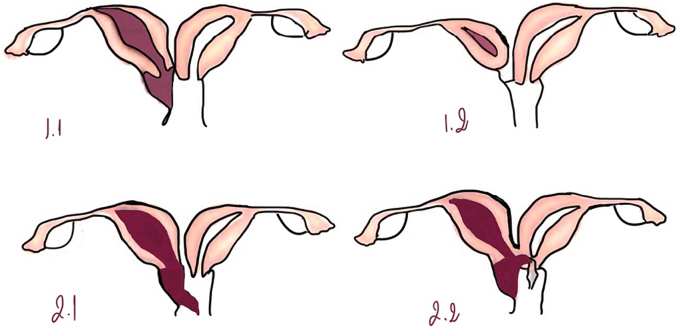
classification of the Herlyn-Werner-Wunderlich.

This syndrome has two types depending to Lan Zhu et Al. (classification 1 and 2) according to the complete or partial obstruction of the hemivagina as the clinical details linked to each type are distinctly different [7]. Each classification has two types: (Fig. 6)

o In classification 1.1, the hemivagina is completely obstructed and the both uterus do not communicate. (as is in current case).
o In classification 1.2, the hemivagina is completely obstructed; the cervix behind the septum is atretic.
o In classification 2.1, there is a link between the two hemivaginas.
o In classification 2.2, the hemivagina is obstructed at 100 %, and a little communication exists between the duplicated cervices.

The syndrome of HWW is often discovered at puberty, the diagnosis of this syndrome is often delayed because of the regular menstruation with cyclic dysmenorrhea for the first few months, but because of the obstruction of the hemivagina, there is a retention of menstrual flow leading to Hematocolpos, complicated over the months by pelvic pain and dysmenorrhea. When the vaginal septum is incomplete symptomatology is not very revealing and complications appear before.

Pelvic pain is the most common symptom followed by an abdominal mass and pressure symptoms. [8,9] Early detection and prompt surgical resection of the obstructing vaginal septum can relieve pain, has a good prognostic outcome with preservation of fertility and prevent further complications like endometriosis, pyohematocolpos, pyosalpinx, or pelviperitonitis. Long-term risk of hypertension, proteinuria, and chronic kidney disease in children with one functioning kidney [6,10].

If this syndrome is suspected, the diagnosis is simply and it can be made by ultrasound, computed tomography and/or MRI.

Ultrasound is usually the first imaging study used for diagnosing HWWS, due to being widely available, convenient and radiation-free, especially in younger patients.

However, MRI has been shown to be the gold standard in imaging of Müllerian anomalies as it has an excellent evaluation of soft tissues and can provide clear details of the uterine cavity and associated anomalies [4,11]. Early diagnosis and management are important to resolve obstructive symptoms and prevent the development of endometriosis from retrograde menstruation. The definitive treatment of Herlyn-Werner-Wunderlich syndrome is surgical excision of the vaginal septum and patients can have a normal sexual life [12].

The vaginoscopy approach with incision of the vaginal septum is a convenient, minimally invasive bloodless and safe for treating HWWS that preserves the integrity of the hymen in adolescents.

Whereas the traditional approach was more aggressive and more painful, often involving the use of a scalpel, vaginal valves and retractors, the vaginoscopy approach is better suited to the needs of the patient because the septal resection can preserve hymen integrity and No vaginal suture is needed [13].

In cases of complication, ipsilateral hysterectomy is recommended because resection of the septum would not relieve symptoms. Successful pregnancy in a previously Herlyn-Werner-Wunderlich syndrome has been reported. [13]

Postoperative follow-up is based essentially on a clinical and ultrasound examination, vaginal stenosis or recurrence of the hematocolpos is a postoperative possibility.

Patients' fertility is usually preserved, albeit with a high percentage of miscarriages, threatened premature birth and intrauterine growth restriction. [14]

## 4 Conclusion

The difference exist between clinical manifestations and age of onset may differ concerning types of Herlyn–Werner–Wunderlich syndrome (HWWS). Ultrasound is highly recommended for screening and MRI, for diagnosis. Early diagnosis and surgical treatment can avoid further complications.

Vaginoscopy resection of vaginal septum is the best option, for young girls who wish to preserve the hymen, it's a virginity conserving surgery, especially in a cultural context, this treatment is a very simple and minimally invasive, it reduces operating time, pain and possible complications.

We report a case report of this malformation following the recent scare guidelines. [15]

### 4.1 Patient perspective

Our young patient was living in endless pain, and had unfortunately missed several courses.

Today she can finally live a normal life with which she is satisfied.

## Consent

A consent was obtained from the patient to publish this case report and accompanying images, written informed consent was obtained from the patient's parents/legal guardian for publication and any accompanying images. A copy of the written consent is available for review by the Editor-in-Chief of this journal on request.

## Ethical approval

Ethics approval has been obtained to proceed with the current study. Consent to participate not applicable.

## Funding

No funding or grant support.

## Author contribution

Farah FLISSATE, Aziz BAIDADA: performed surgery, paper writing and editing.

Najia ZERAIDI, Amina LAKHDAR: literature review, Supervision.

Farah FLISSATE, Hounaida MAHFOUD, Youssef ESSEBAGH: Manuscript editing, picture editing,

## Guarantor

Farah FLISSATE, Gynaecology-Obstetrics and Endoscopy Department, Maternity Souissi, University Hospital Center IBN SINA, University Mohammed V, Rabat, Morocco

Email: Flissate.farah@gmail.com

## Declaration of competing interest

The authors declare that they have no competing interests relevant to the content of this article.
